# Complementary and alternative medicines (CAMs) and adherence to mental health medications

**DOI:** 10.1186/1472-6882-14-93

**Published:** 2014-03-10

**Authors:** Edel Ennis

**Affiliations:** 1 School of Psychology, University of Ulster, Northland Rd, L.Derry BT48 7JL, Northern Ireland

**Keywords:** Adherence, Medication, Mental health, Complementary and alternative medications (CAMs)

## Abstract

**Background:**

Medication regimes are often poorly adhered to, and the negative consequences of this are well recognised. The dynamics underlying non-adherence are less understood. This paper examines adherence to prescription medications for mental health difficulties in relation to the use of complementary and alternative medicines (CAMs). This was based on suggestions that within medical pluralism, CAMs may reduce adherence to conventional prescription medications for reasons such as their further complicating the medication regime or their being perceived as a substitute with less adverse side effects than conventional prescription medications.

**Methods:**

Data used was from the National Comorbidity Study Replication (NCS-R), specifically those 1396 individuals who reported taking a prescription drug for mental health difficulties within the last 12 months and under the supervision of a health professional. This subsample was selected due to their being the only subgroup questioned regarding their medication adherence. Other demographic and health factors were also considered.

**Results:**

The use of complementary medicines alongside the conventional medicines bore no significant relation to odds of reporting adherence versus non adherence. Ethnicity and medication count were significant predictors of adherence versus non-adherence.

**Conclusions:**

The above findings are discussed from the point of both promoting the use of CAMs and increasing health professionals’ understanding of the dynamics underlying adherence, or the lack thereof, and subsequently informing interventions to reduce the problems associated with this issue in terms of increased health care needs and reduced quality of life.

## Background

Adherence^a^ is “the extent to which a patient’s behavior coincides with medical or prescribed health advice” [1]. It averages only 50% among patients suffering chronic diseases (physical or mental) [2,3]. As a widespread major obstacle to treatment effectiveness, patient quality of life, and a burden and demand on the health care system and the family, health professionals need to understand and address the dynamics of adherence, or the lack thereof [2]. This paper examines adherence amongst those who reported taking a prescription drug for mental health difficulties, and the extent to which this relates to the use of complementary and alternative medications.

Several theoretical models have been applied to understanding medication adherence [4,5]. However, there there is a large degree of concordance across the majority of these theoretical models. They are primarily ecologically based, focusing on both social and cognitive factors, and on factors spanning the illness and its medication/treatment regime, the person/patient himself/herself, and the interactions between the health care provider and the patient [5-9]. This means that effective interventions to increase adherence are individualized, long-term, complex multilayered matters and many meet with limited success [3,6-8,10]. Using relevant theoretical frameworks to guide us towards an understanding of the factors associated with adherence versus non adherence, is essential to enhancing our understanding of the matter and subsequently developing effective interventions to reduce the problem [11].

An interesting area within the treatment of illnesses (both physical and mental) is the enduring popularity of medical pluralism. Medical pluralism is “the use of more than one medical system or the use of both conventional and complementary and alternative (CAM) therapies” [12]. CAM encompasses any healing practice that does not fall within the realm of conventional medicine, but these practices are referred to as complementary medicine when used in conjunction with mainstream techniques, or alternative medicine when used instead of it [13]. Using the theoretical frameworks that have previously been applied to understanding adherence, the present study examines the proposition that medical pluralism in the form of the use of complementary medicines may relate to non-adherence to conventional prescription medications for mental health difficulties [14].

Given the enduring popularity of medical pluralism or the use of CAMs [12,15], the limited attention devoted to the relation between their use and adherence to conventional prescription medications is somewhat surprising [16]. For example, a review validating the Information Motivation Strategy (IMS) model of adherence does not discuss the possible role of CAMs at all [6]. Similarly, a review of patient self-reported barriers to adherence to antihypertensive medications using the World Health Organization multidimensional adherence model makes no mention of the possible importance of CAMs or medical pluralism [5]. Various categories of barriers are discussed in terms of health system barriers, patient related barriers, therapy related barriers, socioeconomic related barriers and condition related barriers. While many of these could be indirectly related to the possible role of CAMs and medical pluralism, the article in itself does not do this explicitly.

Existing evidence has been inconsistent about whether CAMs use relates to adherence to prescription medical regimes [7,9,14,16,17], or perceived effectiveness of conventional medicines [14]. Discrepancies in findings may be due to differences in the illness studied, the measurement of adherence, the definition and form of CAM employed, or even the sample or culture studied. To contribute to overcoming this caveat, the current study uses a large scale American random sample (NCSR) to examine seeks to examine whether use of complementary and alternative medicines (CAMs) may relate to non-adherence to conventional prescription medications for mental health difficulties.

The primary reason suggested for a link between these two domains has been the proposition that CAMs may further complicate the medication regime and thus reduce adherence to conventional prescription medications [9]. It is known that the more medications a patient uses, the less likely the patient is to be adherent to a medication regimen [9]. It has been suggested that CAM use requires substantial time and energy, and may be difficult for patients to incorporate into their day-to-day life [16,17]. As a result, CAM users may feel logistically and/or psychologically overburdened and find it necessary to sacrifice parts or their entire prescribed treatment regimen to continue practicing CAM [16]. Thus, while some patients may use these alternative medications as complementary to their conventional prescription medications, some may use them as an alternative to their conventional prescription medications. Secondly, it has been noted that for many illnesses, patients may often dislike taking conventional medications due to unpleasant side effects [16]. This may increase the likelihood that patients may discontinue taking their prescribed medications and replace them with CAM therapies which often have less frequent or less bothersome side effects [16,18]. These issues as barriers to adherence can easily be aligned with either the categories identified by the World Health Organisation multidimensional adherence model, or the IMS adherence model.

The current focus on the use of CAMs in relation to adherence to conventional treatments of mental health difficulties is also a welcome addition to current literature from the point of view that although interest in the topic is increasing, relatively little research has examined the use of CAM among adults with serious mental illnesses (SMI) [14]. Although there has been some research conducted amongst those experiencing serious mental illnesses (SMI) e.g. bipolar sufferers [14], the majority of evidence has been amongst those experiencing physical illnesses such as hypertension [5,7], asthma [19], irritable bowel disease [20]. Non-adherence to medications for mental disorders remains high [11,14], with rates being lower than those documented in relation to physical illnesses [11]. This increased interest is due to the fact that rates of CAM use among those experiencing mental illness are relatively high [11,14]. This is possibly due to the historical difficulties with conventional treatments for mental illness in terms of the speed with which they show results suggesting ineffectiveness for patients, and also continued distress due to the persistence of symptoms of the mental illness and also side effects of the medication, although side effects for newer medications are slightly less intrusive than they were for older medications [11,14]. Patients with psychiatric illness typically have great difficulty following a medication regimen, but they also have the greatest potential for benefiting from adherence [11].

Integration of treatment approaches is essential as CAM therapies offer benefits when used appropriately and are popular amongst lay people and health professionals alike [9,14,21]. Health professionals must understand the relationship between CAMs and prescription medication adherence [7]. Overall, the current study expands existing knowledge concerning personal adherence to prescribed medications for mental health issues in terms of how it relates to medical pluralism. This group of individuals (those taking prescription medications for mental health difficulties) is focused upon as it was desirable to use the NCSR due to it being a large scale random representative sample, and within this dataset only those taking prescription medication for mental health difficulties are questioned concerning their adherence. It is hypothesized that the use of CAMs may predict adherence versus non adherence to prescription medications for mental health difficulties. In trying to understand adherence, alongside CAMs, the role of demographics such as sex, age, poverty index, education, ethnicity, marital status, and health factors such as health status and medication count, psychiatric morbidity and chronic physical health will also be considered. These factors will be incorporated into the analysis as they are personal and health factors which have already been identified by the World Health Organization (WHO) as influential within adherence in general [2]. WHO outline some other factors may play a role within adherence [2], however the aforementioned are those demographic and health factors for which information is available for within the NCSR. “A better understanding of populations at greater risk of non-adherence, and possible barriers to the appropriate medication use, can offer targets for future interventions” [22].

## Method

The NCS-R assesses the prevalence and correlates of mental disorders in a nationally representative sample of the US [23]. A full report of the methods employed within the NCS-R can be found at Kessler et al. [23]. The Human Subjects Committees of both Harvard Medical School (HMS) and the University of Michigan approved the recruitment, consent, and field procedures of the NCS-R [23]. These ethical guidelines are compliant with the Helsinki Declaration on ethical principles for medical research involving human subjects.

### Samples

The overall NCS-R sample consisted of 9,282 individuals. The subsample selected for analysis were those who reported that in the last 12 months, they had taken prescription drugs for mental health difficulties, with this consumption being under the supervision of their health professional (N = 1396). These were selected due to their being the only subgroup questioned regarding their medication adherence. An additional 144 individuals reported also taking these same medications, but not under the supervision of a health professional. This subgroup was not included in analyses due to their lack of medical supervision and their not being questioned concerning adherence.

The sample (the medication group) is 15% of the overall NCSR sample. Demographics are outlined in Table 1 and are taken from the screener section of the NCS-R, namely sex, age, marital status, education, ethnicity and poverty index. Many of the variables are already coded within the dataset, but recoding of the information concerning age, ethnicity and poverty index followed the format of Kessler et al. [24].

**Table 1:** Logistic regression using sample characteristics, health profile and complementary medicines as predictors of adherence versus non adherence

	***N***	***B***	***Wald Chi-square***	***odds ratio***	***95% CI for odds ratio***	
					***Lower***	***Upper***
Use of CAMs (ref = none)	660		1.66			
Only non-pharmacological CAMs	225	-.05	.05	.95	.64	1.43
Only herbal remedies / supplements	34	.56	1.45	1.75	.70	4.35
Both non-pharmacological and herbal remedies / supplements	103	-.06	.05	.94	.53	1.65
Drug type (ref = sleeping pills/sedatives)	80		8.01			
Anti-depressant medications	431	.19	.41	1.21	.68	2.15
Tranquilizers	60	-.31	.52	.73	.31	1.71
Amphetamines or other stimulants	5	.38	.11	1.47	.16	13.61
Anti-psychotic medications	10	-.40	.20	.67	.11	3.91
Other mental health medicine	79	-.65	2.58	.52	.24	1.15
Multiple medication types	357	-.07	.05	.93	.51	1.70
Chronic physical health conditions (ref = absent (N = 88) v present)	934	.10	.12	1.11	.62	1.98
Psychiatric morbidity (ref = no disorders)	298		1.60			
1 disorder	209	-.25	1.16	.77	.49	1.23
2 disorders	158	.06	.05	1.06	.64	1.77
3 or more disorders	357	-.08	.14	.92	.59	1.44
Medication count		-.06	4.59*	.94	.88	.99
Sex (ref = male (N = 296) v female	726	-.01	.00	.99	.70	1.40
Age 18-29 years (ref)	148		4.76			
30-44 years	327	-.01	.00	.99	.56	1.73
45-59 years	345	.00	.00	.99	.55	1.79
60 years plus	202	-.52	2.12	.60	.30	1.20
Ethnicity (ref = non-hispanic white)	858		6.60			
Non-hispanic black	72	.48	1.93	1.62	.82	3.21
Hispanic	57	.73	4.87*	2.07	1.08	3.94
Other	35	.34	.43	1.40	.51	3.83
Marital status (ref = married or cohabiting	524		1.80			
Previously married	316	-.12	.36	.89	.60	1.31
Never married	182	.25	.99	1.29	.78	2.11
Family income (ref = low)	245		.97			
Low average	278	.19	.63	1.21	.76	1.93
High average	288	.01	.01	1.01	.62	1.65
High	211	.13	.23	1.14	.67	1.95
Education 0–11 years (ref)	161		.18			
12 years education	280	.04	.02	1.04	.63	1.72
13-15 years education	329	-.04	.02	.96	.56	1.63
16 or more years education	252	-.05	.03	.95	.54	1.69

Key; *p < .05; ref = reference group.

### Materials

#### Pharmacoepidemiology

Medication use and adherence were assessed within the pharmacoepidemiology section of the NCS-R. Participants were asked two questions concerning their consumption of prescription medicines in the past 12 months for mental health difficulties. The first was a specific question “In the past 12 months, did you take any of the following types of prescription medications under the supervision of a doctor, for your emotions or nerves or your use of alcohol or drugs?”, which was followed by five sets of specific examples (sleeping pills or other sedatives (such as ambien or sonata); anti-depressant medications (such as prozac or zoloft); tranquilizers (such as xanax or ativan); amphetamines or other stimulants, (such as ritalin or dextroamphetamine); anti-psychotic medications, (such as haldol or risperdal)), with participants being required to respond yes / no to each category. This was followed by a more general question “Did you take any type of prescription medicine in the past 12 months for problems with your emotions, substance use, energy, concentration, sleep, or ability to cope with stress?”, with participants again responding yes / no. Participants who indicated a yes answer to any of the above questions were subsequently asked whether this medication consumption was under the supervision of their health professional. Only those who indicated that their medication consumption was under the supervision of a health professional (N = 1396) were examined in the current study, as only these were interviewed in detail concerning adherence to their medications^b^ Whilst this was the general criteria for inclusion within the sample, within the binary logistic regression analysis, these six categories of drug type considered separately.

Specifically, “People do not always take their medication as they are supposed to. Think of a typical month when you took (med) in the past 12 months. How many days out of 30 did you typically either forget to take it or take less of it than you were supposed to take?” Participants were asked about their adherence to three medications. However, Pearson’s correlation coefficient showed a strong correlation coefficient between the measures of adherence to each of the three medications (r = 51, r = .52, r = .72, all p < .001). Although there is slight variation in the strength of these correlation coefficients, all are statistically significant. This is therefore taken as suggesting that non-adherence to one medication was accompanied by similar degrees of non-adherence to other medications, and thus only the first measure of adherence will be used as an overall measure of adherence. All individuals within this subgroup had an adherence score, which could range between 0 and 30 depending on the number of days within the month that they reported forgetting to take their medication. A score of 0 therefore indicated perfect adherence. All other scores (i.e. not taking the medication anything between 1 and 30 days) were regarded as non-adherence. The question on medication count was also taken from this pharmacoepidemiology section.

#### Complementary and alternative medicines (CAMS)

Within the services section of the NCSR, respondents were given a list of commonly used alternative therapies and asked “Did you use any of these therapies in the past 12 months for problems with your emotions or nerves or your use of alcohol or drugs?” For the purposes of the present study, the list of specified therapies were coded as non-pharmacological CAMS (e.g. acupuncture, biofeedback, chiropractic, energy healing, exercise or movement therapy, hypnosis, imagery techniques, massage therapy, relaxation or meditation techniques, self-help and internet support groups, spiritual healing by others) and herbal remedies/supplements (herbal therapy (e.g., St. John’s Wort, chamomile), high dose mega-vitamins, homeopathy, special diets). CAM use was measured following the methodology of Woodward, Bullard, Taylor, Chatters, Baser and Perron [25]. Accordingly, “prayer or other spiritual practices” was not included in the CAM group [25]. The NCSR did include a question on the use of CAMs other than those on the list. This question was omitted from the current analyses as it would not be possible to determine whether it was herbal remedies/supplements or non-pharmacological.

#### Mental disorder status

Mental disorder status was assessed by a structured interview, namely the WHO Composite International Diagnostic Interview (WMH-CIDI) [26]. DSM-IV disorders were examined following the format of the principal leader of the World Mental Health project, of which the NCS-R is a component [24]. Disorders considered were those outlined by Kessler [24], and included diagnoses from the domains of mood disorders, anxiety disorders, impulse control disorders and substance disorders [24]. The criteria of Kessler [24] were also used to consider the matter of psychiatric morbidity in that individuals were grouped as to whether they had no disorder, one disorder, two disorders, or three or more disorders [24].

#### Chronic conditions of physical health

Within the chronic conditions of the NCSR, participants were asked whether or not they had ever had arthritis or rheumatism, chronic back or neck problems, frequent or severe headaches, any other chronic pain, seasonal allergies like hay fever, a stroke, heart attack, heart disease, high blood pressure, asthma, tuberculosis, chronic lung disease like COPD or emphysema, diabetes or high blood sugar, ulcer in the stomach or intestine, HIV infection or AIDS, epilepsy or seizures, or cancer. Participants were coded in terms of whether or not they indicated that they had any of these conditions. It was not possible to determine whether the participant still had or still received any treatment for their chronic condition as this information was not obtained for all illnesses.

#### Analyses

The specified hypothesis was examined using binary logistic regression. The minimum level for statistical significance was .05 within all analyses. As data is based on part 1 and part 2 variables, for the binary logistic regression analysis, data was weighted based on weight 2 within the NCSR data set [23]. Frequencies reported within Table 1 are those which SPSS included in the analysis after weighting.

## Results

Of those who reported taking prescription medications for mental health difficulties under the supervision of a health professional (N = 1396), 710 individuals (50.9%) reported perfect adherence (missing their medications on 0 days per month), 496 individuals (35.5%) reported non adherence (missing their medications on anything between 1 and 30 days per month) and adherence information was missing for 190 individuals (13.6%). Binary logistic regression assessed the hypothesis. Adherence versus non adherence to the prescription medications for mental health difficulties was the outcome variable. The use and type of complementary medications, elements of personal health profile (drug type, medication count, the absence versus presence of a chronic physical health condition, and psychiatric morbidity) were used as predictors, as were sample demographics (age, sex, ethnicity, marital status, education, and poverty index).

A test of the full model with all 11 predictors against a constant only model was not statistically significant (χ^2^ (29, N = 1022) = 41.22, p > .05), indicating that that predictors do not make a significant contribution to the prediction of adherence versus non-adherence. However, classification was unimpressive with a success rate of 64%, which is practically identical to the 63% classification rate indicated based on block zero of the model (i.e. the chances of correctly predicting whether an individual was adherence or non-adherent if we knew nothing about the predictor variables). In order to consider the rate of classification as impressive, the accuracy rate of classification of the model including the predictor variables would need to be a substantial improvement on the classification ability of the model without predictors. Similarly, Nagelkerke *R*^*2*^ yielded a value of .08, indicating that the combined predictors only explained 8% of the variance in adherence / non-adherence.

Based on the Wald statistics, results partially rejected the hypothesis. CAMs (use or type), drug type, chronic physical health conditions (absence versus presence), psychiatric morbidity, nor medication count, significantly predicted the odds of reporting non-adherence versus adherence (Table 1). Sex, age, marital status, poverty index, and education were also non-significant predictors of non-adherence versus adherence (Table 1). Ethnicity was related to medication adherence in that in comparison with those who identified themselves non-Hispanic white background, those of Hispanic background were twice as likely to report non adherence (Table 1). The only other significant predictor was medication count in that as ones medication count increased, the likelihood of reporting adherence decreased and the likelihood of reporting non adherence increased (Table 1). This was partially supportive of the initial hypothesis.

## Discussion

The current results agree with the prevalence of non-adherence to conventional prescription medications [2,3], even when these are being taken under the supervision of a health professional. Results were partially supportive of hypothesis one in that only ethnicity and medication count were significant predictors of adherence versus non adherence to conventional prescription medications for mental health difficulties. Health factors such as drug type, psychiatric morbidity, absence versus presence of a chronic physical health condition, and demographic factors such as sex, age, poverty index, education and marital status, bore no relation to the prediction of adherence versus non adherence. Furthermore, similar to some studies [9,14], the current results show no relation between the use of CAMs and adherence to prescription conventional medications. This was regardless of the type of CAM used (non-pharmacological treatments or herbal remedies / supplements).

This is noteworthy given the enduring popularity of medical pluralism or the use of CAMs [12,15]. This finding contributes to allaying fears that the potential benefits of integrating CAMs may be undermined by their reducing adherence to conventional prescription medications. Integration of treatment approaches is essential as CAM therapies offer benefits when used appropriately and are popular amongst lay people and health professionals alike [9,14]. This current results provide some evidence to refute the suggestion that patients using CAM may be doing so as an alternative to conventional medicine as opposed to complementing treatment regimens [16]. It must however still be acknowledged that these findings are in contrast to others which have shown use of CAM to be associated with reduced adherence to conventional prescription medications [9,16,17]. Further in-depth research is necessary in terms of actually interviewing people, and possibly considering different illnesses (across both the physical and mental sector) independently.

Care must also be exercised in view of the fact that although CAM was not related to adherence versus non adherence, in agreement with earlier research [9], increased medication count was a significant predictor of non-adherence. Health professionals must be mindful to keep patients medication regime as uncomplicated as possible. Health professionals should also question patients regarding any complementary therapies that they use and ensure that the patient has a plan as to how both regimes can be effectively integrated into their life style as opposed to the burden of one domain resulting in the sacrificing of the other domain [16]. This is necessary as many patients do not tell their physicians and thus explicit dialogues is necessary as provider awareness of their patient’s use of CAM therapies may be important to ensuring that patient preferences are recognized to help ensure continued engagement in conventional treatment [9,14]. Such explicit conversations are also necessary to avoid situations such as drug–drug interactions, drug substitutions with CAM, and treatment noncompliance, particularly among persons with serious mental illnesses [14]. Further research on this domain is necessary given that although increased medication count was associated with non-adherence, the use of herbal remedies / supplements was not directly related to non-adherence to conventional prescription medications.

Based on their suggested importance [2], demographic factors (sex, age, education, ethnicity, marital status and poverty index) as well as elements of personal health (drug type, medication count, psychiatric morbidity, and absence versus presence of a chronic physical illness) were included in the model. Findings regarding medication count have already been discussed. The lack of association between physical health and adherence to conventional medications could be said to be somewhat curious. It is possibly because the data does not examine the degree of the health problem and the impact on the activities of daily living. It does not allow distinction between acute illness and chronic illness, or even consideration of whether the illness is being effectively controlled by medication or even in remission. It could be tentatively suggested that with severe acute physical health problems, the likelihood of adherence may go up. With chronic problems, adherence may possibly go down.

Medication adherence was unrelated to sex, age, education, marital status or poverty index. However, findings suggested that ethnicity was related to medication adherence in that in comparison with those who identified themselves non-Hispanic white background, those of Hispanic background were twice as likely to report non adherence. Presence versus absence of a chronic physical illness bore no significant relation to non-adherence. BMI was not included as a marker of physical health. This was because in the realm of mental health problem, whilst appearing unhealthy, an increased BMI could be associated with adherence to psychotropic medication. Many (but not all) of these are associated with weight gain.

### Limitations

Medication adherence was unrelated to psychiatric morbidity. Psychiatric morbidity did not appear to compromise their ability to manage personal medications. However, a limitation must be acknowledged in that within each category, the range of severity is potentially large. For instance, an affective disorder may manifest itself as a mild depressive episode at one end of the spectrum and as a bipolar I disorder at the other. Severity is accounted for to some degree by controlling for clinical diagnosis. Whilst useful and informative, the current data format may not show the entire picture and further in-depth study may be required. Medication count may constitute a marker for severity of illness or an expression of conventional medicines not working, however this proposition could not be tested. Kessler et al. [24] outline criteria under which clinical disorders may be coded as mild, moderate or severe [24]. However, the algorithms necessary to do this are only available to the World Mental Health (WMH) team.

It is also noteworthy that there were individuals who were taking a medication for a mental health difficulty under the supervision of a health professional, yet did not meet neither the DSM nor the ICD criteria for the presence of any mental health difficulty (mood disorder, anxiety disorder, substance disorder, nor impulse disorder). The question had asked whether they had taken a prescription drug for problems with their emotions, nerves, their use of alcohol or drugs energy, concentration, sleep, or ability to cope with stress, with this consumption being under the supervision of their health professional. Whether they have a DSM or ICD diagnosis outside the four mentioned categories warrants attention.

In particular, the unique issues of psychosis require consideration. This matter was somewhat problematic within the current dataset. Although anti-psychotics were amongst the list of specified medications, information concerning diagnosis of psychosis is somewhat vague. For the mental health difficulties considered in this paper, participants were asked a large range of questions and these responses were evaluated by trained researchers against the DSM and ICD criteria. However, with regard to psychosis, individuals were asked whether or not they had ever had a series of experiences, if they had consulted their health professional concerning this, and if so, what had diagnosis had their health professional applied. This is obviously reliant on the accuracy of the participants memory, and given the difference in this and how the diagnosis of the other mental health difficulties was applied, the current paper considered the information available insufficient to actually apply a diagnosis of psychosis.

The current study simply provides a one point in time picture. The data assessed adherence to supervised medications for mental health difficulties, and whether this is comparable to adherence for physical health difficulties is unknown. Some evidence suggests that interview based self-reports of medication adherence are not concordant with electronic measures of actual adherence [27]. However, such measures have been used in scientific research in peer reviewed academic journals and have received positive evaluations within reviews where the conclusion has been that no one method should be considered as the “gold standard” [11].

The current data focuses on people who self-reported taking medication. For future research, it could be said to be important to include people who were offered medication but declined to accept it as it could be suggested that this is the ultimate example of non-adherence. Other important issues to consider may include ethnicity, family relations, as well as severity and chronicity of mental health problems. With regard to the NCSR, the matter of chronicity of illness is complicated. For each mental illness, timing of first episode is often (although not always) asked of the participant. However, there may be confusion concerning first clinical episode versus first appearance of milder subclinical symptoms.

## Conclusions

Further in-depth research on the practical applications of these findings and the additional influential factors is needed given the small proportions of variance explained, and the lack of an increase in correct classification. It is extremely concerning that non adherence to prescription medications for mental health difficulties is such an extensive problem, and even though a vast array of factors were considered in the current analysis, it was not possible to explain a significant proportion of the variance in adherence versus non-adherence. This could be considered as a warning of just how complex the issue is, and the difficulty that health professionals face in trying to understand the matter. The possibility must also be considered that that factors explaining adherence to medications for pure physical health difficulties may differ somewhat from those factors which explain adherence to medications for mental health difficulties.

To conclude, poor medication adherence is an extensive problem [2,3] which needs examined in terms of social and personal vulnerability (behaviors, cognitions and perceptions) [5-9]. A matter of concern is that despite the breadth of the factors included in the current analysis, it was not possible to significantly explain the variance in adherence versus non-adherence. Vulnerability factors to non-adherence may include ethnicity and increased medication count. Further research is necessary, but the current findings suggest that efforts to understand the dynamics underlying non adherence to conventional prescription medications should move away from studying the domain of use of CAMs. Factors for health professionals and researchers to consider in the future may include the domains of family burden and caregiver role. Evidence from some small studies suggests that in their endeavor to provide effective care for their care recipient, caregivers may actually compromise their own self-care [28]. “A better understanding of populations at greater risk of non-adherence, and possible barriers to the appropriate medication use, can offer targets for future interventions” [23]. However health profession councils also need to support health care providers in designing and delivering patient targeted interventions [29]. Many health providers need help to work on their accepting their pivotal responsibility for promoting adherence and believing that they influence it, with only a minority routinely assessing adherence in the recommended manner [7,29]. Many health professionals lack training and confidence on how to effectively assess this within a consultation, fearing intrusion of privacy or appearing as challenging the patient [29].

## Endnotes

^a^The current paper consciously uses the term ‘adherence’ rather than ‘compliance’ in recognition of the importance of the interaction between the health professional and the layperson.

^b^It is acknowledged that these “three medications” may be different for different people and adherence to medication may vary as function of whether the medication is perceived as important or providing them with relief from complaints as opposed to limiting future risks for health outcomes. However, all medications reported in this section would be for the treatment of mental health difficulties as opposed to physical health difficulties (although their function may be to address a physical issue that is a secondary symptom of a primary mental health difficulty.

## Competing interests

The author declares no conflict of interest or competing interests.

## Authors’ contributions

No other authors have made any contribution to manuscript preparation.

## Pre-publication history

The pre-publication history for this paper can be accessed here:

http://www.biomedcentral.com/1472-6882/14/93/prepub
